# Environmental Health Research Recommendations from the Inter-Environmental Health Sciences Core Center Working Group on Unconventional Natural Gas Drilling Operations

**DOI:** 10.1289/ehp.1408207

**Published:** 2014-07-18

**Authors:** Trevor M. Penning, Patrick N. Breysse, Kathleen Gray, Marilyn Howarth, Beizhan Yan

**Affiliations:** 1 Center of Excellence in Environmental Toxicology, Perelman School of Medicine, University of Pennsylvania, Philadelphia, Pennsylvania, USA; 2 Division of Environmental Health Engineering, Department of Environmental Health Sciences, Johns Hopkins Bloomberg School of Public Health, Baltimore, Maryland, USA; 3 Center for Environmental Health and Susceptibility, University of North Carolina at Chapel Hill, Chapel Hill, North Carolina, USA; 4 Lamont-Doherty Earth Observatory of Columbia University, Palisades, New York, USA

## Abstract

Background: Unconventional natural gas drilling operations (UNGDO) (which include hydraulic fracturing and horizontal drilling) supply an energy source that is potentially cleaner than liquid or solid fossil fuels and may provide a route to energy independence. However, significant concerns have arisen due to the lack of research on the public health impact of UNGDO.

Objectives: Environmental Health Sciences Core Centers (EHSCCs), funded by the National Institute of Environmental Health Sciences (NIEHS), formed a working group to review the literature on the potential public health impact of UNGDO and to make recommendations for needed research.

Discussion: The Inter-EHSCC Working Group concluded that a potential for water and air pollution exists that might endanger public health, and that the social fabric of communities could be impacted by the rapid emergence of drilling operations. The working group recommends research to inform how potential risks could be mitigated.

Conclusions: Research on exposure and health outcomes related to UNGDO is urgently needed, and community engagement is essential in the design of such studies.

Citation: Penning TM, Breysse PN, Gray K, Howarth M, Yan B. 2014. Environmental health research recommendations from the Inter-Environmental Health Sciences Core Center Working Group on Unconventional Natural Gas Drilling Operations. Environ Health Perspect 122:1155–1159; http://dx.doi.org/10.1289/ehp.1408207

## Introduction

Unconventional natural gas drilling operations (UNGDO) (which include the processes of hydraulic fracturing and horizontal drilling) in tight shale formations to extract natural gas, create jobs, provide a potential route to energy independence, and may increase national security through less dependency on foreign oil (IHS Global Insight 2011). The burning of natural gas produces less nitrogen oxides (NO_x_) and carbon dioxide (CO_2_) than the burning of coal or oil and produces negligible amounts of sulfur dioxide and mercury, and thus is a cleaner fossil fuel [U.S. Environmental Protection Agency (EPA) 2013a, 2013b]. However, concerns have been raised about the environmental and public health impacts of UNGDO (Union of Concerned Scientists 2013). The industry describes the technology as being well established and safe (American Petroleum Institute 2014). By contrast, some advocacy groups have serious environmental health concerns and suggest a moratorium on UNGDO until we learn more (e.g., Physicians for Social Responsibility 2012).

UNGDO have concentrated where large formations of shale exist, for example, the Barnett Shale in Texas, the Utica Shale in Ohio, and the Marcellus Shale in Pennsylvania [U.S. Geological Survey (USGS) National Assessment of Oil and Gas Resources Team and Biewick 2013]. Together, these and other shale gas resources have provided a significant energy resource. For example, the Marcellus Shale contains > 84 trillion ft^3^ of natural gas, which would be sufficient to meet the energy needs of the United States for 2–4 years (USGS 2011). However, in areas conducting UNGDO, there have been incidents of water contamination (Jackson RB et al. 2013), worker exposure to levels of silica dust that exceed OSHA (Occupational Safety and Health Administration) standards (Esswein et al. 2013), and reports of health effects among community residents (Bamberger and Oswald 2012; McKenzie et al. 2014). Because of these issues, some states (e.g., New York) have a moratorium on UNGDO, whereas other state legislatures have considered passing strict regulations on the industry (Pless 2011).

In addition, the need for crystalline silica (frac sand) for use in the hydraulic fracturing process has prompted expansion of mining operations in the upper Mississippi watershed (Wisconsin, Minnesota, and Iowa). This expansion has become a contentious issue in communities because of environmental degradation, lost income from tourism, and risk to respiratory health (Wisconsin Department of Natural Resources 2012).

On the basis of the level of drilling activity in the Marcellus Shale, the Center of Excellence in Environmental Toxicology (CEET), an Environmental Health Sciences Core Center (EHSCC) at the University of Pennsylvania, felt an obligation to address the public health impact of UNGDO on Pennsylvania’s citizens. The CEET recognized that UNGDO will be part of the energy landscape of the future and that credible science is needed to determine their safety in order to establish evidence-based decision making. The CEET realized that the environmental health concerns related to UNGDO could best be addressed by scientists with complementary expertise working together. Concurrently, several Community Outreach and Engagement Cores of the EHSCC identified the growing concerns of citizens and the lack of health-related information. This led to the formation of the Inter-EHSCC Working Group (for a list of members, see Appendix 1). A search in PubMed (http://www.ncbi.nlm.nih.gov/pubmed) using the search term “hydraulic fracturing” identified 111 citations at the time of writing this article. Only a handful were peer-reviewed studies on environmental health, and many are cited here. In addition, the working group considered reports by government and health agencies and nonprofit organizations, as well as reports from the gas and oil industry. This led to several unanimous recommendations.

## Discussion

*Recommendations for research on water contamination*. Groundwater could become polluted due to casement failures and infiltration from soil and surface water during UNGDO. Surface water has the potential to be contaminated by *a*) leakage from wastewater impoundments, *b*) incidents during the transport of wastewater, and *c*) inappropriate discharge from wastewater treatment plants (U.S. EPA 2011a; Warner et al. 2013). Wastewater consists of the initial flow-back water and the produced water, which itself is a mixture of spent hydraulic fracturing chemicals and contaminants, including total dissolved solids that exceed levels found in sea water, aromatic hydrocarbons, heavy metals, and naturally occurring radioactive materials that may leach from the shale (Ferrar et al. 2013b; International Association of Oil and Gas Producers, 2002; Rowan et al. 2011).

In Pavillion, Wyoming, in 2009, the U.S. EPA found evidence of groundwater contaminated with benzene, xylenes, gasoline range organics, diesel range organics, and total volatile hydrocarbons in shallow wells that lie above 169 gas-producing wells that were hydrofractured. The pollution was attributed to the 33 nearby surface pits used to store drilling waste water (Jackson RE et al. 2013; U.S. EPA 2011b). The USGS resampled the area and confirmed these findings (Wright et al. 2012). However, there were still disputes about whether UNGDO were the source of groundwater contamination because of the lack of baseline water quality measurements (American Petroleum Institute 2012). Inter-EHSCC Working Group recommendation: Baseline groundwater quality data should be collected before drilling begins and be monitored over the lifetime and abandonment of the gas-producing well.

Lack of detailed information about the chemicals injected into the shale formations and the composition of the flow-back water makes it difficult to determine whether water quality is affected. A complete inventory of chemical usage, which can exceed > 80 additives (Stringfellow et al. 2014), is currently unavailable. The FracFocus Chemical Disclosure Registry (http://fracfocus.org/), a voluntary database of chemicals used in hydraulic fracturing fluid developed by the industry, provides necessary data to map chemical usage by some wells but not all. This represents the first step in determining whether water quality may be affected on a well-by-well basis. Unfortunately, many of the chemicals in use are proprietary, and the flow-back and produced water can also contain other contaminants, such as polycyclic aromatic hydrocarbons (PAHs) and naturally occurring radioactive materials. For example, in the Marcellus formation, the level of radioactivity in the produced water was reported to be many times higher than allowable for discharge to the environment (Rowan et al. 2011). Inter-EHSCC Working Group recommendation: To determine whether UNGDO affect water quality, the chemicals used in the hydraulic fracturing process must be fully disclosed so that they can be correlated with measurements of ground and surface water pollutants; the composition of the hydraulic fracturing fluid and the produced water must also be analyzed for hazard identification.

There is a need for sensitive and specific early-warning indicators that groundwater has been contaminated. Such indicators would allow researchers and site managers to predict whether UNGDO affect water quality. Suitable indicators would be chemicals derived from UNGDO that have fast rates of transport and can be detected easily in field settings. Candidate indicators are methane, ethane, propane, chloride, sodium to chloride ratio, and chloride to bromide ratio. Jackson RB et al. (2013) reported that concentrations of methane, ethane, and propane in the Marcellus region of Pennsylvania were higher in homes located < 1 km from drilling sites than in homes farther away. Distance to gas wells was found to be a significant determinant of hydrocarbons in drinking water. However, in some private wells, concentrations of methane in the drinking water were elevated prior to fracturing (Vidic et al. 2013; Warner et al. 2012); thus, methane concentrations may not be the best indicator. An increase in the ratio of ethane to methane, propane to methane, and chloride to other major anions (e.g., nitrate) could be used as warning indicators of groundwater contamination. Alternatively, a unique inert tracer could be added to the hydraulic fracturing fluid. Inter-EHSCC Working Group recommendation: A validated specific and sensitive indicator of early groundwater contamination be identified and universally adopted.

Knowledge of the fate and transport of pollutants as well as groundwater hydrology under the influence of pressure changes during and after hydraulic fracturing is required *a*) to determine whether pollutants can migrate to private or public drinking wells, *b*) to identify early-warning indicators, and *c*) to estimate the transit time of target pollutants and identify suitable remediation strategies. Interaction between the pollutant and particle phase determines the speed of pollutant transport and whether the pollutants can reach drinking water wells. Groundwater moves slowly, typically in the range of meters per year, depending on characteristics of the aquifer and hydraulic gradients (USGS 2013). Pollutants that can travel to wells within the span of years are those that are persistent, have high solubility, and are less particle reactive. Pollution of surface water (e.g., spills of hydraulic fracturing fluid and discharge from wastewater plants) would move faster (in meters per second) and can be affected by reactions between pollutants and the particle phase (USGS 2007). Inter-EHSCC Working Group recommendation: Research should be performed to elucidate the fate and transport of ground and surface water pollutants under hydraulic fracturing conditions.

Assessment of effluent contaminants from wastewater treatment plants discharging Marcellus Shale waste in Pennsylvania showed that barium, strontium, bromides, chlorides, and total dissolved solids exceeded the maximum contaminant level for drinking water (Ferrar et al. 2013b). In 2011, the Pennsylvania Department of Environmental Protection requested that drilling companies stop disposing wastewater by this method at 15 facilities (Pennsylvania Department of Environmental Protection 2011). These findings suggest that municipal wastewater treatment plants are unable to deal with contaminants from the produced water and that water quality from these plants needs to be monitored if these plants are to be used for this purpose (Ferrar et al. 2013b). Inter-EHSCC Working Group recommendation: The effluent from a range of wastewater treatment plant technologies should be assessed to determine the effectiveness of the technology.

There is a lack of knowledge of the toxicological properties of the hydraulic fracturing chemicals alone or in complex mixtures. However, the proprietary nature of these chemicals indicates that this may never be known. Knowledge of the chemical additives would enable risk characterization, that is, the identification of no observed adverse effect levels (NOAELs) or lowest observed adverse effect levels (LOAELs) for each chemical and the reference doses for which exceedance may cause harm in humans. However, because the chemicals are used in a complex mixture, toxicological studies will be required on the mixture itself. The mixture will also have to be fractionated in order to determine which chemicals or group of chemicals are the most harmful. In this approach, compounds can be grouped by chemical similarity or by similarity in toxicological effects (European Commission Scientific Committee on Consumer Safety 2011; Meek 2011). Subfractions could be triaged using high throughput cell-based screens for genotoxicity, mutagenicity, cytotoxicity, and endocrine-disrupting properties. Components identified for further study could then be used in acute, intermediate, and chronic exposure studies in rodents to identify toxic end points. Inter-EHSCC Working Group recommendation: Fundamental research on the toxicology of the individual constituents of hydraulic fracturing fluid and the resultant complex mixture should be performed.

*Recommendations for research on air pollution*. Hazardous air pollutants related to UNGDO include *a*) silica dust from mining, handling, transport, and disposition of sand (Esswein et. al. 2013); *b*) diesel emissions from delivery trucks, compressor stations, power generators, and drilling rigs (Benbrahim-Talla et al. 2012); *c*) volatile organic compounds in the flow-back and produced water as well as their reaction with NO_x_ to increase ground level ozone (Kemball-Cook et. al. 2010); and *d*) fugitive gas emissions during the production phase and from well ruptures (Allen et al. 2013). Increased local and regional ambient air pollution has been associated with intensive gas extraction regions (Eaton 2013; Kargbo et al. 2010; Pétron et al. 2012). However, the spatial and temporal release of these pollutants will depend on the intensity of the various sources (emission rates) and their locations (e.g., frac sand mines, frac sand transfer stations, and truck transport routes to and from the well pad; and the proximity of well pads, produced-water containment ponds, and waste impoundments to each other and to affected communities) is not well characterized and needs to be addressed. Inter-EHSCC Working Group recommendation: Ambient and occupational air-quality should be measured at active drilling sites and be compared with baseline measurements in adjacent areas without UNGDO.

PM_2.5_ in diesel exhaust (from > 2,200 trucks per drill head) can exacerbate respiratory illness, and chronic exposure to diesel exhaust may increase the risk of lung cancer (Benbrahim-Tallaa et al. 2012). That assessment of lung cancer risk was based on diesel exhaust emissions that predate the 2007 new emission standards. It is not known how the diesel emissions associated with UNGDO meet these new standards, and this should be determined. Using geographic information system modeling, levels of diesel pollutants could be related to truck traffic patterns in order to identify local hot spots and regional impacts that could be mitigated. Inter-EHSCC Working Group recommendation: The impact of diesel emissions on local air quality should be determined.

Airborne emissions containing ambient pollutants from UNGDO may affect indoor residential air quality when they penetrate indoor environments. Data on indoor as well as outdoor UNGDO-related pollutant concentrations are thus needed. Residential air quality for people living adjacent to frac sand transfer stations or truck transport routes should be compared with that of people living away from such sources in order to generate a comparison to baseline data. Inter-EHSCC Working Group recommendation: Residential indoor air quality data for homes that are potentially affected by UNGDOs should be compared with unaffected homes.

Coal-fired power plants can emit greenhouse gases: CO_2_, sulfur oxides, NO_x_, mercury, trace metals, and products of incomplete combustion such as PAHs (U.S. EPA 2013a). However, few studies have compared levels of air pollution produced by these power plants with levels produced by a field of natural gas wells. Only when these measurements are made will it be possible to evaluate the potential health risks and benefits of UNGDO compared with the use of coal. Inter-EHSCC Working Group recommendation: The impact of UNGDO on air pollution should be compared with emissions produced by coal-fired power plants.

*Recommendations for epidemiologic research*. Prospective longitudinal epidemiologic studies to measure the association between health effects with proximity to UNGDO can be conducted only if the health end point is known. A good starting point would be to use health outcome and utilization data from national and local databases to associate illness and health-care visits with proximity to UNGDO. The working group recognized that baseline data in control communities, by census block, in which UNGDO is not occurring is key to identifying differences that could become end points in a prospective epidemiologic study. For example, using health outcome data, McKenzie et al. (2014) observed an association between well density and proximity of natural gas wells within a 10-mile radius of maternal residence with the prevalence of congenital heart defects in newborns. Epidemiologic studies should also include environmental sampling and/or biomonitoring of exposures to demonstrate a dose- or exposure-dependent association with the end point(s) being measured. Studies should include occupational exposure and vulnerable populations (e.g., pregnant women, children, the elderly, individuals with asthma).

Carrying out an epidemiologic study linking water pollution from UNGDO to health effects is problematic because the contaminants are not fully known and because of the variability of drinking water sources, preexisting water quality, chemicals used, temporal relationships, and underlying hydrology. Exposure assessment would require measurement of suspected contaminants in communities with UNGDO and in adjacent communities without UNGDO where baseline data could be obtained. Biomarkers of exposure to water contamination could include measured blood concentrations of heavy metals (e.g., lead) and biomarkers of volatile organic compounds (e.g., benzene metabolites). These biomarkers are short-lived, but measurement of longer-lived biomarkers (e.g., serum albumin–benzoquinone adducts) is an alternative (Rappaport et al. 2011). To support a causal relationship between water pollution and health effects, a plausible mode of action would need to be identified. Inter-EHSCC Working Group recommendation: An environmental epidemiology study should be performed to determine whether an association exists between health outcome data and water quality in private drinking wells in communities with and without hydraulic fracturing.

An epidemiologic study linking air pollution to health effects is less problematic than is one related to water pollution because the air pollutants are known and the disease end points recognized. Recent studies by the National Institute for Occupational Safety and Health (NIOSH) have documented excessive crystalline silica exposures at UNGDO sites (Esswein et al. 2013). In addition, McKenzie et al. (2012) estimated that the increased exposures to airborne hydrocarbons in Garfield County, Colorado, resulted in a small increase in cumulative cancer risk of 10 new cases in 1 million individuals living within 0.5 mile of gas-producing wells. Short-, intermediate-, and long-term exposures of workers and residents to air pollutants resulting from UNGDO and exacerbation of underlying respiratory illness (e.g., asthma, chronic obstructive pulmonary disease) and cardiovascular disease (e.g., ischemic heart disease, dysrhythmias, heart failure, cardiac arrest) may be more sensitive indices of adverse health effects than cancer incidence (Pope et al. 2004). Inter-EHSCC Working Group recommendation: An environmental epidemiologic study should be performed to determine whether air pollution associated with UNGDO increases the incidence of respiratory illness and cardiovascular disease.

*Recommendations on integrating community perspectives in environmental health research*. Health impacts and stressors are perceived to exist in communities with UNGDO (Bamberger and Oswald 2012; Ferrar et al. 2013a). Given that elements of a property owner’s control may cease once UNGDO begins, these perceptions are consistent with an involuntary risk model, based on a lack of control of an unknown hazard with little opportunity for independent verification of safety (Sjöberg 2000; Slovic 1987). Issues raised by UNGDO are similar to those of communities impacted by other industrial operations in early stages of development: Limited data on health indicators and health impacts make it difficult to identify and track health effects as well as the latency of effects. Limited to no baseline or monitoring data makes it challenging to track environmental health impacts over time.

Community-based participatory research (CBPR) provides a framework for engaging community members in research and has been effectively applied to a number of environmental health problems (Minkler et al. 2006; O’Fallon and Dearry 2002). CBPR goes beyond sharing research results with community members to creating meaningful opportunities for community participation in all stages of research (i.e., project scoping, data collection, analysis, and dissemination). Inter-EHSCC Working Group recommendation: CBPR principles should be embraced in designing and conducting studies on environmental and health impacts of UNGDO so that a range of community perspectives are addressed. All stakeholders (individual/community/industry/advocacy groups/decision makers) should be engaged early to foster multidirectional communication and accountability.

CBPR requires that study results be communicated to the communities in a timely manner (Chen et al. 2010). Emmett et al. (2009) recommended a “Community-First” communication model, which shares research findings with the affected community before publishing them in scientific literature in order to empower the community by reducing information disparities. Inter-EHSCC Working Group recommendation: Communities should be engaged in determining the most effective ways to disseminate research findings, and dissemination of aggregated data should be timely and transparent.

Because the potential exists for lower-income communities to bear a greater burden of any negative outcomes of UNGDO, it is important to engage members of the community whose health and environment may be disproportionately impacted by this activity (Adams 2012). Inter-EHSCC Working Group recommendation: Health disparities due to UNGDO should be addressed in the design of human studies.

Impacted communities demand transparency in the research process, especially with respect to who is funding the research. This, in part, stems from mistrust of industry and efforts to limit access to either information on chemicals used in hydraulic fracturing or on-site environmental testing results (International Energy Agency 2012). Inter-EHSCC Working Group recommendation: The sources of funding for research on the environmental health impacts of UNGDO need to be openly disclosed.

In two small, rural communities in Pennsylvania and New York, Brasier et al. (2011) reported that the infrastructure and social services were overwhelmed by the onset of UNGDO and the concomitant population influx. In addition, in a review of medical issues related to UNGDO, Saberi (2013) described barriers faced by family physicians, who often are unable to counsel their patients about the effects of environmental exposures related to hydraulic fracturing because of limited training in occupational and environmental medicine. Inter-EHSCC Working Group recommendation: The impact of rapid industrialization on public health and health-care services, including training needs of health-care providers, should be evaluated.

Communities have identified a need to understand the regulations that govern UNGDO. Only six states allow health-care providers access to proprietary chemical constituents, and four of the six require the health-care provider to sign a confidentiality agreement restricting disclosure to others (McFeeley 2012). Denying health-care providers access to chemical information for patient care purposes is unprecedented, as is restricting disclosure to individuals who are exposed. Inter-EHSCC Working Group recommendation: Research should be conducted to determine how existing regulations affect reporting of environmental health consequences of UNGDO in order to enable the development of more health-protective regulations.

Risk perceptions encompass cognitive evaluations of the likelihood of harm as well as emotional responses. Risks that are most feared are those that are unknown, experienced involuntarily, potentially catastrophic, and risky for future generations—all factors that are in play with UNGDO (Sjöberg 2000; Slovic 1987). Having an understanding of the nature of community perceptions on UNGDO will inform risk communication and risk management. It will also determine whether credible sources of information are being used to set view points and will identify critical information gaps. Inter-EHSCC Working Group recommendation: Research should be conducted on risk perception, including the effects on community polarization.

## Conclusions

The research recommendations of the inter-EHSCC working group are similar to those proposed by others (Goldstein et al, 2014; Shonkoff et al 2014; Union of Concerned Scientists 2013) with one significant difference: We advocate for a CBPR approach in communities affected by UNGDO. Implementation of these recommendations would inform the debate on the potential environmental health affects of UNGDO and lead to decisions by individuals, communities, agencies, and industry that would protect human health. Implementation requires dedicated funding sources that are insulated from conflicts of interest so that the science generated is trustworthy. One trusted model is federal agencies funding research that is conducted at academic institutions. Oversight by a single organization would avoid duplication of effort and unnecessary expenditure of resources. There should be harmonization of study designs, data collection, and analytical procedures, which may require a data coordination center that could also assess data quality and missing data. There should also be a publicly available data repository so that all stakeholders, including industry and communities, can access data, and appropriate firewalls and limited access should be in place for patient- or population-based health data. Implementation of these recommendations would permit a risk assessment of UNGDO, enabling decision makers to identify and reduce the most serious environmental health threats.

**Appendix 1:** Inter‑EHSCC working group members

Name	Affiliation
Working group members
Regina SantellaSteven ChillrudBeizhan Yan	Columbia Mailman School of Public Health
Douglas DockeryAnn Backus	Harvard School of Public Health
John GroopmanPatrick Breysse	Johns Hopkins Bloomberg School of Public Health
Max Costa	New York University
Joseph BeckmanKim Anderson	Oregon State University
Helmut Zarbl	Rutgers University
Shuk-mei HoErin Haynes	University of Cincinnati
Peter ThorneDavid Osterberg	University of Iowa
David PeteringJeanne Hewitt	University of Wisconsin–Milwaukee
James SwenbergKathleen Gray	University of North Carolina at Chapel Hill
Trevor M. PenningGeorge GertonMarilyn HowarthRey PanettieriPoune Saberi	University of Pennsylvania
Tom GasiewiczKatrina KorfmacherDavid Rich	University of Rochester
Frank GillilandAndrea Hricko	University of Southern California
Cornelius ElferinkSharon PetronellaJohn Sullivan	University of Texas Medical Branch
Terry Kavanaugh	University of Washington
Affiliate member
Lisa Mckenzie	University of Colorado School of Public Health
Ex officio members
Leslie Reinlib Liam O’Fallon	National Institute of Environmental Health Sciences
